# Book Review: An Introduction to Behavioral Endocrinology (5th Edition)

**DOI:** 10.3389/fnhum.2018.00161

**Published:** 2018-04-23

**Authors:** Martin G. Köllner

**Affiliations:** Human Motivation and Affective Neuroscience Lab, Department of Psychology, Institute of Psychology, Friedrich-Alexander University Erlangen-Nürnberg (FAU), Erlangen, Germany

**Keywords:** behavioral endocrinology, biology, hormones, reproductive behavior, sex differences, social behavior

The fifth edition of *An Introduction to Behavioral Endocrinology* (Nelson and Kriegsfeld, 2017) is tasked with the ambitious goal of delivering an introduction to a very broad and highly inter-disciplinary field of study that is of genuine interest to neuroscientists, especially the ones studying social behaviors related to for example affiliation and aggression. Generally speaking, this goal can be considered accomplished, as the book provides a comprehensive overview including topics ranging from basic introductory descriptions of hormones, methods and history of the discipline, and the endocrine systems up to a chapter on phenomena as complex as affective disorders. With four of 13 chapters being devoted to sex differences in behavior or reproductive behavior of each sex, respectively, emphasis is placed on sex steroids and their contribution to sex differences. However, sex differences are a core topic in psychology (Halpern, 2012) and neuroscience (Cahill, 2006) and so this decision is justified. Later chapters discuss parental and social behavior, homeostasis and behavior, biological rhythms, and stress. A multi-chapter treatment of sensorimotor function and cognition like in *Behavioral Endocrinology* (Becker et al., 2002) has not been included as a coherent separate part of the book (but see for example a—however brief—treatment of sex differences in cognitive abilities in chapter 4), but the final chapters on learning and memory and on affective disorders, respectively, make up for this and should be especially relevant for psychologists.

Regarding presentation of the content and aptness for undergraduate courses, the book contains many illustrations and graphs that should enhance readability and comprehension especially for students. Every chapter starts with learning goals for reading and ends with a summary of the main points and questions for discussion. The chapters are accompanied by online supplemental materials which encompass for example videos, animations, and links to further readings for students. The quantity of these materials varies by chapter, with for example chapter 13 containing only one item and chapter 8 around 20. From a didactic perspective, *An Introduction to Behavioral Endocrinology* can thus be recommended.

Looking for possible drawbacks of the present work, first it is—as the cover correctly states—an introduction, not an edited book in which every chapter is authored by specialized experts on the given topic. Thus, after having gained an overview on a given topic, researchers in behavioral endocrinology will still feel the need to consult additional references on a focused area of expertise. The book provides some aid in this regard, as chapters are also accompanied by suggested readings. Second, to readers who—like myself—have a background in psychology, the book may still be easily accessible and does not require excessive endocrine and/or biological previous knowledge. Nevertheless, the authors suppose that psychology majors have some basic understanding of biological aspects before being instructed with the book (acquired for example in a biopsychology course; p. XVI). In addition, deliberate emphasis on a comparative perspective—chosen despite being criticized by readers from the field of psychology (as the authors acknowledge, p. XV)—may render some parts of the book, like the sexual behavior of male rodents (chapter 5) or the vaginal cytological assay in rats (chapter 6), a tedious read for psychologists, with little relevance to behavioral studies in human neuroscience at first sight.

However, the latter drawback is a small one—and admittedly, the comparative sections do broaden one's understanding of mammal homologies if one has exclusively studied human behavior previously, and sections regarding birds or reptiles illustrate the function of hormones in other vertebrates. The book is balanced in this regard, as, on the other hand, genuinely psychological topics like learning and memory in chapter 12 are explained from the very basic concepts (e.g., associative learning, habituation) on to readers of other disciplines who thus may also gain insights beyond their primary area of expertise.

In sum, the book is a must-read for anyone who plans to study the vibrant field of behavioral endocrinology. As a final note, to put it bluntly, there simply is no comparable book out there, as the other authoritative reference on the topic, *Behavioral Endocrinology* (Becker et al., 2002), never went beyond its second edition and thus has inevitably missed out on the last 16 years of development of the field.

## Author contributions

MK wrote the manuscript and is the sole contributor of this work.

### Conflict of interest statement

The author declares that the research was conducted in the absence of any commercial or financial relationships that could be construed as a potential conflict of interest.
